# Menstrual Fluid Factors Mediate Endometrial Repair

**DOI:** 10.3389/frph.2021.779979

**Published:** 2021-12-21

**Authors:** Lois A. Salamonsen

**Affiliations:** Centre for Reproductive Health, Hudson Institute of Medical Research and Department of Molecular and Translational Science, Monash University, Clayton, VIC, Australia

**Keywords:** menstrual fluid, endometrial repair, Re-epithelialization, scar-free repair, endometrium

## Abstract

Menstruation is a process whereby the outer functionalis layer of the endometrium is shed each month in response to falling progesterone and estrogen levels in a non-conception cycle. Simultaneously with the tissue breakdown, the surface is re-epithelialized, protecting the wound from infection. Once menstruation is complete and estrogen levels start to rise, regeneration progresses throughout the proliferative phase of the cycle, to fully restore endometrial thickness. Endometrial repair is unique compared to tissue repair elsewhere in the adult, in that it is rapid, scar-free and occurs around 400 times during each modern woman's reproductive life. The shedding tissue and that undergoing repair is bathed in menstrual fluid, which contains live cells, cellular debris, fragments of extracellular matrix, activated leukocytes and their products, soluble cellular components and extracellular vesicles. Proteomic and other analyses have revealed some detail of these components. Menstrual fluid, along with a number of individual proteins enhances epithelial cell migration to cover the wound. This is shown in endometrial epithelial and keratinocyte cell culture models, in an *ex vivo* decellularized skin model and in pig wounds *in vivo*. Thus, the microenvironment provided by menstrual fluid, is likely responsible for the unique rapid and scar-free repair of this remarkable tissue. Insight gained from analysis of this fluid is likely to be of value not only for treating endometrial bleeding problems but also in providing potential new therapies for poorly repairing wounds such as those seen in the aged and in diabetics.

## Introduction

The endometrium, the inner lining of the uterus, provides the maternal support for embryo development and undergoes remarkable remodeling on a cyclical basis. In humans and a few other species including old world primates, the endometrium develops more extensively during each menstrual cycle than in other placental mammals. In particular the process of decidualization, which provides the basis for the decidua of pregnancy, is initiated whether or not the cycle is one in which conception occurs. As this process is not reversible, the endometrium is shed in the process known as menstruation, then fully repaired and reconstructed during the subsequent cycle. Importantly, in contrast to most adult wounds, the endometrium repairs very rapidly (days) without scarring (otherwise seen only in fetal tissues) and occurs over 400 times during a woman's reproductive lifespan. Most aspects of menstruation are discussed elsewhere in this volume. The focus of this article is the role of menstrual fluid (MF) in the scar-free repair of the endometrium.

### Menstruation: Endometrial Shedding

Understanding the cellular and molecular events of menstruation provides insights critical to understand the likely composition of menstrual fluid.

During menstruation, the outer functional layer of the endometrium is shed in a piecemeal manner, with breakdown and rapid repair occurring simultaneously at adjacent sites (1, 2). Shedding is finely controlled so that while most or all of the functional layer is shed, the basalis remains *in situ*. re-epithelialization occurs rapidly but it is from the basalis that the full thickness endometrium subsequently regenerates (3). What is not yet known is the mechanism preventing degradation of the basalis although it has been proposed that the horizontal network of glands forming a “rhizome-like” layer may be limiting (4).

Menstruation is considered to be a highly regulated inflammatory response to progesterone withdrawal. Initiation of menstrual events occurs in the decidualized endometrial stromal cells, which express progesterone receptors and hence sense hormone withdrawal. These cells initiate a sequence of inter-dependent inflammatory events including nuclear translocation of NF-κB, a transcription factor that regulates both innate and adaptive immune responses. NF-κB signaling induces the progressive production of many inflammatory cytokine and chemokine mediators, increased prostaglandin synthetic enzymes and production of pro-inflammatory prostaglandins (5). The released chemokines recruit and activate leukocytes (predominantly granulocytes) into the endometrium. Variable numbers and types of immune cells are present in the functional layer throughout the menstrual cycle, but are in low abundance until the pre-menstrual stimulus that initiates a massive and highly selective influx of leukocytes. Perimenstrually, these comprise up to 50% of the total cells population. 6–15% of all nucleated cells in the stromal compartment of the functional endometrial layer are neutrophils, the same abundance as for macrophages (CD16^+^) and uterine natural killer (NK) cells (CD56^+^/CD16^−^) but eosinophils, mast cells (both 3–5%) and *T* lymphocytes (1–2%) are less common [review; (6)]. Importantly, these cells are phenotypically different from their counterparts in peripheral blood indicating the effects of the local microenvironment. For example, production of active elastase is much reduced and alpha1-anti-trypsin highly elevated in endometrial neutrophils compared with peripheral blood neutrophils (6). Together these leukocytes, many detectable in activated forms, establish an inflammatory cascade which results in tissue breakdown. These complex phenotypic changes in a highly dynamic physiological setting in women, severely limit investigation of their individual functions. It is also possible that cellular senescence, particularly of the decidualized stromal cells, plays a role; this has been recently described in human endometrial assembloids (7) but any contribution to menstruation remains to be established.

Importantly for the tissue breakdown at menstruation, each non-migratory cell in the functionalis epithelial, stromal, decidualized stromal cells (5, 8) also directly or indirectly responds to progesterone withdrawal by releasing an array of proteolytic enzymes including matrix metalloproteinases, plasminogen activator family members and other molecules, with considerable interactions occurring that initiate self-activating cascades. These can be between products of both resident cell and leukocytes. For example, *in vivo*, endometrial-derived immune cells produce a wide range of enzymes important for other cell activation (e.g., degranulation of eosinophils induced by neutrophil elastase), or molecular processing such as conversion of latent to active matrix metalloproteinases by elastase or cathepsin G. These combined actions result in degradation of the extracellular matrix (ECM) and tissue breakdown (9, 10). Tissue shedding during menstruation is piecemeal; fragments of endometrial tissue can be found in menstrual fluid (MF) along with single endometrial cells, blood, and ECM debris.

### Endometrial Repair

Endometrial repair is uniquely scar-free, is initiated almost immediately as shedding starts and is complete by the time bleeding ceases (up to 8 days) (11). Degrading tissue and re-epithelializing sites are seen adjacent to one another in the menstruating tissue by histology and scanning electron microscopy (1, 2) and once bleeding ceases (when re-epithelialization is complete), regeneration of the entire tissue thickness is initiated. The rapid re-epithelialization serves to protect the tissue from bacterial invasion as it regenerates. Initially, re-epithelialization is observed as migration of epithelial cells from the exposed stumps of glands and these can be seen by scanning electron microscopy to expand outwards to meet similar cells from other glands or those migrating from any intact remaining epithelium (2, 12). Repair of damage to transverse sub-epithelial endometrial arterioles within the stroma and to spiral arterioles, which can be severely injured during tissue breakdown, occurs concomitant with re-epithelialization. However, full regeneration of the endometrium is primarily if not entirely from stem/progenitor cells present in the basalis layer (which is not shed). This regeneration requires estrogen action, and is complete by the time of ovulation, ~ 14 days after the start of menstruation, and 9 days following cessation of bleeding and full re-epithelialization (13). Data has indicated that mesenchymal to epithelial cell differentiation (EMT) contributes to restoration of the luminal epithelium at least in mice (14, 15); however, recent *in vivo* cell fate-tracing studies in mice have found no evidence for EMT in endometrial repair [(16), reviewed in (13)]. Evidence for an EMT contributing to human endometrial repair should be further examined.

Most adult wounds repair with scar formation, which may impair function and inhibit further growth whereas repair of the endometrium (and of wounds in fetal tissue), is scar free. There are also differences between wounds in the adult oral cavity and elsewhere in the body (17), largely due to unique mediators in saliva. Wound healing in general involves a complex interplay between numerous cell types, cytokines, mediators and the vascular system. Wounding in all tissues is accompanied by an influx of inflammatory cells, starting with neutrophils and their local release of chemokines that attract other leukocytes to the wound site. These cells together release a range of mediators and cytokines that promote re-epithelialization, angiogenesis and thrombosis. The fibroblasts in turn secrete ECM components that provide scaffolding for the cellular events (18). The scar tissue that forms in most adult tissues, results from the formation and extension of fibrous tissue (fibrogenesis) derived primarily from stromal cells (19). However, the healed endometrium is without obvious fibrosis. Furthermore, while repair of most wounds takes 4–6 weeks, repair of the endometrial surface is generally complete within 5 days.

Since in most tissues, stromal cells are the major effectors of scarring, it must be assumed that endometrial stromal cells derived from stem/progenitor cells during endometrial regeneration are differently programmed. Given that re-epithelialization to cover the endometrial surface is so rapid, the stem/progenitor cells are likely brought into play more quickly than in other tissues. Interestingly, transforming growth factor (TGF)β1, a factor that strongly promotes the myofibroblast phenotype, is elevated in menstrual fluid compared with peripheral blood and could theoretically act on endometrial stromal cells *in vitro* to differentiate them into myofibroblasts (19). Since this does not occur, it must be that *in vivo*, either the TGFβ1 must be non-functional, or other regulatory stimuli must prevent such differentiation to prevent scarring. Importantly, menstrual fluid, added to cultures of stromal cells of adipose and dermal origin, suppresses their transition to myofibroblasts as it does with endometrial stromal cell cultures, supporting this contention (19). However, the active suppressive factor/s remain to be identified.

### Uterine Fluid and Menstrual Fluid

Given that endometrial repair occurs rapidly within a microenvironment of menstrual fluid, evidence is now emerging that menstrual fluid contains bioactive factors that promote scar-free repair. What is currently known of these components and their potential actions will be discussed below. However, it is important to set the scene by first considering the composition of uterine fluid which changes throughout the menstrual cycle.

### Uterine Fluid

During the menstrual cycle, a small volume of fluid is always present within the uterine cavity and its components (both soluble and extracellular vesicles) vary between the proliferative and secretory phases and between fertile and infertile women. A number of major soluble components that appear in uterine fluid even outside of menses, are transudated from blood, although this is very selective. Differential protein composition between peripheral blood and uterine fluid was first shown clearly in the 1980's with the advent of two-dimensional gel (2D-DIGE) analyses (20, 21). These studies highlighted proteins specific to uterine fluid and identified differences in fluid composition between the proliferative and secretory phases. Subsequently, 2D-DIGE identified a number of major serum proteins in uterine fluid (human serum albumin, transferrin, immunoglobulins (Ig)G and A, antitrypsin, haptoglobin and hemoglobin), which could be removed prior to further analyses, improving sensitivity for subsequent analysis of the remaining soluble factors (22–24). Such depletion at least doubled the number of proteins that could be identified (24) and which differed between receptive and non-receptive states in fertile and infertile women (23).

Other soluble components of uterine fluid are contributed from peritoneal and tubal fluids, and from endometrial epithelial cell secretions, particularly those of the glands. Uterine fluid proteins, including cytokines and chemokines have been examined using protein array technologies applied to samples taken across the menstrual cycle and between fertile and infertile women (22, 23, 25–27), with >30 cytokines, chemokines and growth factors being identified. These include interleukin (IL)-1β, IL-6, IL-12, IL-17, IL-18, tumor necrosis factor (TNF)α, macrophage migration inhibitory factor (MIF), eotaxin, monocyte chemotactic protein (MCP) 1, interferon-gamma (IFNγ)-inducible protein-10, vascular endothelial growth factor (VEGF), platelet-derived growth factor (PDGF)-AA and chemokine (C-X-C motif) ligand 1–3, all of which are detectable in >90% of samples.

There is no correlation between amino acid concentrations in serum and uterine fluid: 18 amino acids have been identified in human uterine fluid, their concentrations being altered by maternal diet. These include asparagine, histidine, serine, glutamine, valine, isoleucine, and leucine (28). In addition, lipids, a range of metabolites [review; (29)], miRNAs [review; (30)], and small extracellular vesicles (sEV) previously known as exosomes (3, 31, 32), have been identified and/or harvested from uterine fluid obtained from cycling women. These sEVs contain a cohort of miRNAs and proteins, with changes in their proteomes being defined between cycle phases (3, 31).

### Menstrual Fluid

Menstrual fluid (MF) is most often harvested into menstrual cups, a relatively non-invasive and convenient method that can be managed at home. The most common time of collection is on the second day of menstruation, when menstrual flow is maximal. Importantly, MF collection using a menstrual cup has proven to be highly reliable and reproducible between women and between cycles (33). Among the components of MF are debris from tissue breakdown, live cells or groups of cells (epithelial, stromal, vascular) released when the surrounding extracellular matrix (ECM) is degraded, activated leukocytes and their products, endometrial stem cells and extracellular vesicles.

**Figure 1:**
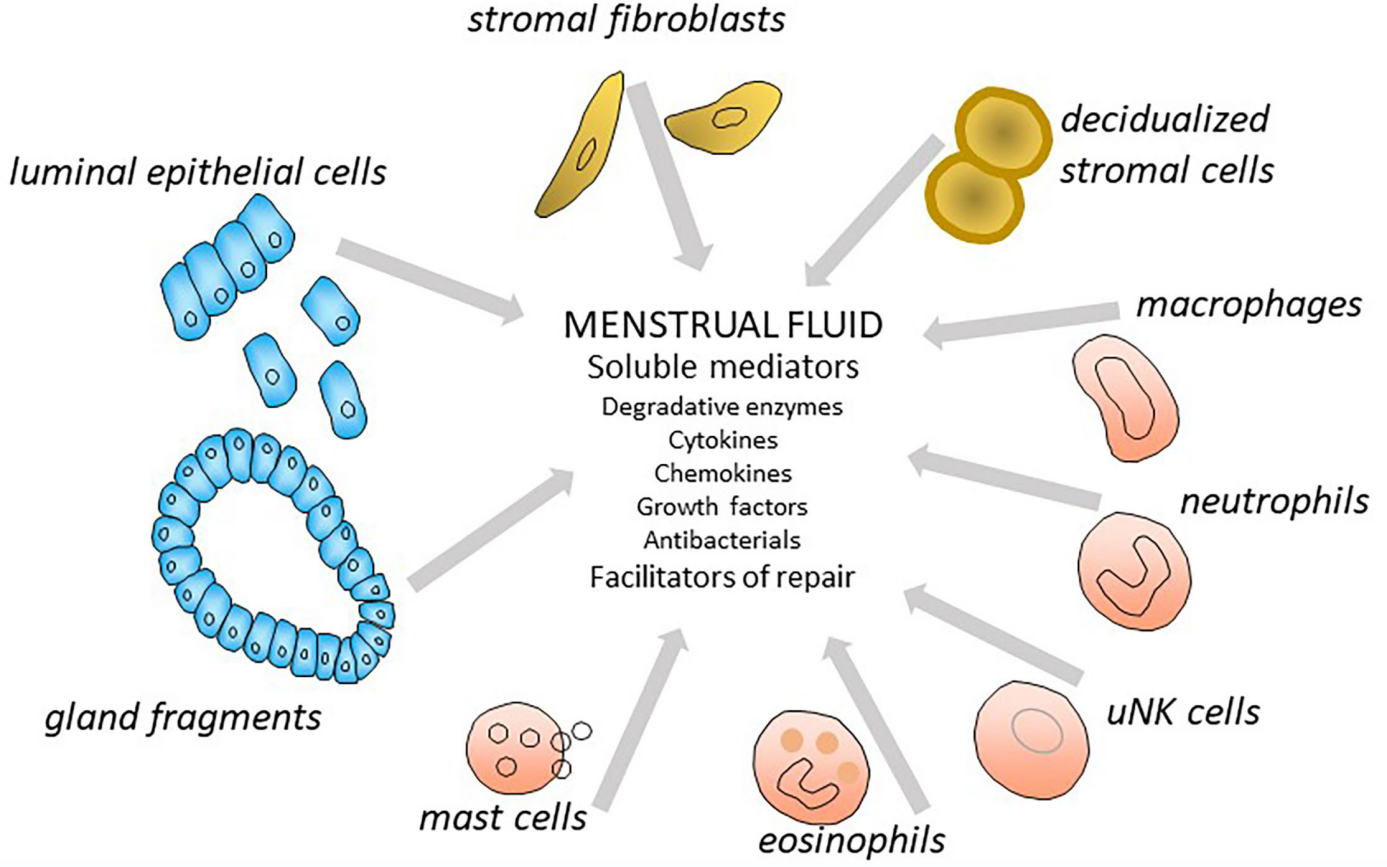
The likely cellular origins of soluble components of menstrual fluid.

Since menstruation is a controlled inflammatory event, resulting in tissue breakdown, MF would be expected to contain many more soluble components than the uterine fluid of the late secretory phase, which immediately precedes menstruation. The additional soluble molecules will be derived from many cellular sources, including endometrial and immune cells (particularly the neutrophils, macrophages, uterine NK and mast cells, that are abundant in the tissue during menstruation), along with intracellular components of degraded cells. These are likely also to contribute to endometrial repair (summarized in Figure 1), a concept supported by reduced uNK cell numbers in late secretory tissue in women with heavy menstrual bleeding (34).

#### Soluble Components of Menstrual Fluid

##### Soluble Contributions From Non-migratory Endometrial Cells

Soluble components of menstrual fluid have been studied in much less detail than those of uterine fluid. Early studies arose from the need to understand how menstrual bleeding is regulated. Uterine haemostasis differs from that in other organs, in that the endometrial haemostatic plugs are morphologically different and very short lived (35, 36). Coagulation and fibrinolytic proteins measured in menstrual fluid supernatant on days 1 and 2 of normal menstruation, showed a virtual absence of thrombin-generating activity and very much higher levels of fibrin-related antigen, active plasmin and plasminogen activator than seen in normal serum, while functionally active α-2-antiplasmin was undetectable (37). Disappointingly this data did not provide a pointer to the mechanisms controlling menstrual blood loss. However, prostaglandins (PG) *F*_2_α and *E*_2_, are also present in menstrual fluid, and levels correlate directly with menstrual blood loss (38). Furthermore, a lack of detectable 9-ketoreductase or 9-hydroxydehydrogenase activity indicates there could be interconversion between the two PGs.

Most reproductive hormones do not show a difference between peripheral venous blood and menstrual plasma. However, while menstrual follicle stimulating hormone (FSH), estradiol-17β and progesterone are likely to arise entirely from the peripheral circulation, prolactin (PRL) levels are elevated only in menstrual blood (39), indicating release into the menstrual flow from mid-late secretory endometrium, where PRL is expressed both in the epithelium and in the decidualized stroma (40).

Recent application of state-of-the-art proteomics techniques (41, 42) to supernatant from centrifuged menstrual fluid (menstrual plasma) vs. matched peripheral plasma have provided extensive lists of proteins specific to menstrual fluid. While both investigators depleted the samples of abundant serum proteins, Evans additionally enriched samples using a combinatorial peptide ligand library (CPLL), along with capture of heparin and fibronectin binding proteins separately on the depleted samples. The most abundant identified proteins from these two studies are listed in Table 1. Many reflect the state of the endometrium in the late secretory phase when pre-decidual changes are evident with concomitant changes in protein production, including insulin growth factor binding protein (IGFBP)-1, matrix metalloproteinase (MMP)9, galectin 3, glycodelin A, glucose transporter (GLUT)1, IL1 and others (24). However, additional proteins not present before menstruation were identified, reflecting induced protease activity from epithelial and decidualized stromal cells, and factors derived from cells released upon tissue lysis (41). VEGF was also significantly elevated in menstrual fluid vs. peripheral plasma.

**Table 1:** Proteins elevated in menstrual fluid vs. peripheral plasma and known to facilitate repair.

**Protein name**	**Known as**	**Previously known actions in repair**	**References**
Neutrophil gelatinase -associated lipocalin/lipocalin-2	NGAL	Promigratory in epithelial cells	*(43)*
			
Epidermal fatty acid binding protein-5	FABP-5	Augments peroxisome proliferator-activator receptor δ in promoting proliferation and survival	*(44)*
Follistatin-related protein 1	FSTL-1	See-saw regulation in wounded skin – inverse relationship with miRNA-198	*(45)*
Macrophage migration inhibitory factor	MIF	Proposed pro and anti-repair actions in skin, probably due to different skin models tested.	*(46, 47)*
Secretory leukocyte protease inhibitor	SLPI	Various roles in cell migration depending upon the system	*(24, 48)*
Human epididymis protein 4	HEP4	migration	*(49)*
S100 proteins	S100A8 S100A9 S100A11	Promote cell migration, but not proliferation Cell motility	*(50, 51)*
Lactotransferrin/lactoferrin	LTF	Promotes skin repair	*(52, 53)*
Stanniocalcin-1	STC1	Angiogenesis	*(54)*
Ninjurin-2	NINJ2	Adhesion protein, promotes cell growth	*(55)*
Neuroblast differentiation-associated protein	AHNAK	migration	*(56)*
Osteopontin	OPN	Cell survival, proliferation, migration	*(57)*
Galectin	Gal1 Gal3	Migration, proliferation Repair	*(58, 59)*
Macrophage inhibitory factor	MIF	Pro-inflammatory antibacterial	*(60)*
Interleukin 8	IL8	Attracts and activates neutrophils	*(60)*
Vascular endothelial growth factor A	VEGF-A	Neo-angiogenesis, Re-epithelialization	*(61)*

*Data derived from (41)*.

More recently, using a custom magnetic Luminex assay, common inflammatory and repair proteins: secretory leukocyte protein inhibitor (SLPI); lipocalin-2 (NGAL); lactoferrin; follistatin-like 1 (FSTL1); and human epididymis protein-4 (HE4), were identified in >60% of menstrual fluid samples analyzed (62), reflecting their previous recognition (41). Interestingly, a negative association between menstrual fluid volume and abundance of some of these proteins (HE4, galectin-1, MIF, SLPI, NGAL, and FSTL1) was revealed following normalization for total menstrual fluid protein (ng/mg). It may be that as menstrual fluid volume increases, other endometrial- or peripheral-derived proteins may similarly increase, thus diluting the proteins of particular interest. Interestingly, many of the menstrual fluid factors listed in Table 1, positively cross reference with those in Senequest (https://Senequest.net/) which contains factors involved in cellular senescence.

Matrix degrading enzymes, including a number of matrix metalloproteinases (MMP), are major players at menstruation, and are released from endometrial epithelial and decidualized stromal cells specifically as progesterone levels fall and also from activated leukocytes (see below). These are accompanied by the release of potential activators and tissue inhibitors of MMPs (TIMPs) which are abundant in endometrial tissue and which together tightly control MMP actions (63, 64). Although only MMP9 was identified in a proteomic analysis of menstrual fluid (41), menstrual serum showed a pattern of MMP activity on zymography different from that of peritoneal fluid while both MMP-7 and MMP-9 were identified by Western blot uniquely in menstrual serum (65). While MMPs are very important for tissue breakdown, they also play roles in tissue repair largely due to their broad protease activities not related to matrix degradation. Some but not all actions on repair, have been validated in individual genetically-modified mouse models including those null for MMP1, MMP8, MMP9, MMP10, and MMP14 (66). In other repair situations, epithelial-derived MMPs facilitate cell migration by affecting cell-matrix adhesion. For example, in mucosal epithelia, MMP7 facilitates re-epithelialization by cleaving different ECM or ECM-associated proteins to affect integrin: matrix adhesion (66). Indeed MMP7-deficient mice have the most impairment of re-epithelialization of any MMP-null mice generated to date and show disturbance of the affinity of integrin α_2_β_1_ cell-matrix interactions (67). In human endometrium MMP7 mRNA is highly increased in epithelial and decidual cells at menstruation and remains throughout the new proliferative phase indicating a role in repair and regeneration (68). Active MMP7 is recruited to the plasma membrane of epithelial cells, thus escaping TIMP inactivation and allowing processing of membrane-associated growth factors needed for epithelial repair and proliferation (69). The involvement of MMPs in endometrial repair remains to be determined.

##### Soluble Contributions From Immune Cells

Mononuclear cells isolated from menstrual blood samples are phenotypically similar to the reported phenotype for biopsy-derived endometrial cells, and distinct from peripheral blood mononuclear cells [PBMC: (33)] although percentages of NK cells are higher and those of *T* cells are lower.

Macrophages, neutrophils, mast cells, and eosinophils, all degranulate upon activation, releasing their soluble contents. Importantly, active forms have been identified during menstruation by virtue of the extracellular immunostaining of granular contents (70–72). These granulocytes have more than one type of granule and there are many similarities in granule morphology, granule content, stimulus for degranulation, and granule trafficking, most of which are not well-understood, particularly in the context of the endometrium. However, it is clear that there is considerable overlap between contents of granules from different sources; for example, eosinophils, neutrophils and macrophages all release matrix metalloproteinase 9 at menstruation (70).

While there is a paucity of information on immune cell products in menstrual fluid that may be relevant to endometrial repair, elsewhere, eosinophils produce a number of growth factors, including TGF-α and -β, fibroblast growth factor (FGF), EGF, PDGF, and VEGF, which participate in angiogenesis and myocardial repair (73). Eosinophils also produce cytokines, in particular IL5 and IL4 which have roles in wound healing and macrophage differentiation. Indeed, mice overexpressing IL5 displayed insufficient production of ECM components and had impaired wound healing. IL4 is essential for differentiation of macrophages toward an M2 phenotype, and regulating myocardial tissue regeneration (74). Uterine NK cells from menstrual fluid produce IFNγ, granzyme B, and perforin, upon stimulation with IL2 and IL15 (33).

Neutrophils contribute to physiological tissue repair, and seem to be necessary for normal healing at least in part by promoting angiogenesis (75). Furthermore, apoptosis of neutrophils after degranulation provides a powerful stimulus for macrophage differentiation into the anti-inflammatory M2 phenotype, through their production of annexin A1, lipocalin, lactoferrin, and cathelicidin. Neutrophil-derived MMP12 also possesses potent pro-resolving properties (74). Anti-bacterial agents within menstrual fluid including lactotransferrin and NGAL may also play a role in post-menstrual endometrial repair.

Mast cell actions are likewise realized through degranulation and secretion of the granules' content of cytokines or production of lipid mediators, depending on the nature of the stimuli received during activation. Relevant to wound repair, during cardiac tissue re-modeling their main function appears to be associated with regulation of fibrous tissue metabolism (76), and they may both enhance and inhibit post-myocardial fibrosis. Their pro-fibrotic properties are mediated primarily by chymase (present in the uterus only in myometrial mast cells) and tryptase (present in endometrial mast cells) (72), which are identified as activators of TGFβ and angiotensin II, well-known promoters of fibroblast activity. Mast cells also produce and secrete anti-fibrotic mediators such as IL10, IL13, CXCL10, and VEGFA (76).

##### Endometrial Stem Cells in Menstrual Fluid

Cells with mesenchymal stem cell properties have been identified in menstrual blood. Following depletion of red blood cells and CD45^+^ leukocytes from menstrual fluid, endometrial stem/progenitor cells including clonogenic endometrial cells, sushi domain containing-2^+^ (SUSD2^+^) mesenchymal stem cells and N-cadherin^+^ (NCAD^+^) epithelial progenitor cells, have been isolated (62, 77, 78), with limited variability across menstrual cycles (62). These cells are not present in peripheral blood. They are generally retrieved from the menstrual fluid as plastic adherent cells and show differences in immunophenotype, proliferation and differentiation capacities from bone marrow-derived mesenchymal stem cells. Since these cells can be reliably purified from menstrual fluid, they may provide a useful non-invasive source of stem/progenitor cells for clinical application. However, isolation protocols and culture conditions must be standardized to maximize their potential. Importantly a serum-free culture protocol has been established that contains a TGFβ receptor inhibitor, that prevents spontaneous differentiation, apoptosis, and senescence of the clonogenic SUSD2^+^ population and enhances their potency (77).

##### Extracellular Vesicles in Menstrual Fluid

Small extracellular vesicles (sEV) previously termed exosomes, are released from all cells. They act as carriers of “cargo” of bioactive molecules including miRNA, proteins and lipids, which they deliver to specific target cells: the phospholipid membranes of the sEV protect the “cargo” from extracellular degradation. Importantly the proteomes of endometrial epithelial cell sEV depend upon the hormonal environment of the cells of their origin (estrogen or estrogen plus progesterone), but are substantially different from those of the cellular proteomes (79). Endometrial-derived sEV are present in uterine fluid (3, 31, 32), although their role in endometrial repair has not yet been examined. A number of pre-clinical studies have evaluated effects of sEVs on the wound-healing process [review; (80)]. For example, in a mouse burn model, sEV derived from human menstrual blood -derived mesenchymal stem cells injected close to the site of injury, enhanced wound closure and increased neoangiogenesis was evident (81). Furthermore, sEV derived from human umbilical cord blood mesenchymal stem cells stimulate regenerative wound healing via TGFβ receptor inhibition (82). Likewise, EVs derived from normal resident lung epithelial cells, appear to possess anti-fibrotic properties, inhibit TGFβ-WNT cross talk and offer a promising anti-fibrotic treatment (83). If similar mediators are contained in sEV in menstrual fluid, this could provide an explanation for the lack of scarring during endometrial repair.

#### Functional Analyses of Menstrual Fluid and Its Soluble Components

The most difficult task following omics analyses is subsequent determination of the likely functions of the large number of identified molecules. In the context of menstrual fluid proteomics, Evans et al., (41), examined actions of the entire soluble fraction of menstrual fluid in a number of biologically relevant repair models. Subsequently, individual fluid molecules, selected for their known function in wound repair were applied at concentrations selected from their measured concentrations in menstrual fluid. Endometrial cell cultures (ECC1 cell line) and keratinocyte cell cultures (HaCatT cell line) were chosen for these studies for their similarities to primary endometrial epithelial and primary keratinocytes. Classic wounding assays were applied to overconfluent cultures which were “wounded” by vacuum suction followed by imaging and analysis using imaging software daily for 3 days. Real-time label-free analysis of adhesion and proliferation using the xCelligence system (ACEA Biosciences, San Diego CA, USA) was also applied to cultures of similarly treated ECC-1 and HaCatT cells. In more physiological assays, *ex vivo* de-epidermized dermis (DED) pre-parations (84) were prepared and cultured for 4 or 8 days in the presence or absence of menstrual fluid. The area of migration of keratinocytes across the DED was quantified, then tissues were processed and embedded for histological examination and the thickness of both cornified and cellular layers were measured. Finally, *in vivo*, a porcine superficial wound model in juvenile pigs, in which the wounds were created by dermatome, was treated with wound dressings containing either peripheral plasma or menstrual fluid. Dressings were changed at days 3 and 5, with simultaneous imaging and quantification of re-epithelialization until day 7 when healing in such juvenile models is generally complete. By 5 days, re-epithelialization was significantly enhanced; wound area was slightly decreased; epidermal thickness was moderately increased; and number of hairs per-section was moderately increased. The latter three did not reach significance in the limited number of wounds approved by the ethics committee.

An important conclusion from these investigations is that factors in menstrual fluid advance the initial migratory phase of healing, a key difference from current skin repair treatments that stimulate epithelial proliferation, while vascular repair agents in menstrual fluid (including VEGF) will likely play roles in the initial repair of the vasculature and subsequent angiogenesis (41). Regrettably, this study did not include menstrual fluid factors with no known role in repair processes. These remain to be tested and may prove a potential “gold-mine” of new treatments for wound repair.

### Endometrial Vascular Repair

Simultaneously with re-epithelialization, rapid repair of the open blood vessels must take place to stop the bleeding, some 5 days after menstruation starts. While experimental evidence for angiogenesis at this time of the cycle is lacking, circumstantial evidence indicates its likelihood. Menstrual fluid contains the potent angiogenic factor VEGF-A (85), (41), and this is markedly reduced in women with menorrhagia (85). Interestingly, in the rhesus macaque, a naturally menstruating primate, blockage of VEGF action with VEGF Trap, a potent VEGF blocker inhibited new blood vessel development and re-epithelialization of the denuded surface during menstruation (61). Further in a mouse model of menstruation, similar blockade of VEGF action delayed repair of the denuded endometrial surface and inhibited new blood vessel development (61). HIF-1α, a transcription factor known as the master regulator of the cellular response to hypoxia, can regulate VEGF by directly binding the VEGF pro-motor at least in macrophages (86) and thus hypoxia, may thus contribute to endometrial vascular repair. Indeed, women with prolonged menstrual bleeding have decreased endometrial HIF-1α during menstruation and the long bleeding period that follows. However, evidence for the presence of hypoxia during menstruation and repair is mixed (87), review: (88), and any role of HIF-1 in endometrial repair needs to be confirmed.

### Endometrial Regeneration After Menses

By the time menses has ceased, the wounded surface is essentially “repaired”, covered by a new luminal epithelium with junctional complexes making a tight protective surface, that shields the underlying tissue from infection. During the next 10 or so days (cycle proliferative phase) and as estrogen levels rise, endometrial thickness and the full cohort of cellular structures, including glands, stroma, vascular structures and extracellular matrix (both interstitial and basal lamina) is regenerated through massive cellular proliferation in the growing functional layer. Current knowledge of this regeneration process has recently been detailed (13) and will not be further discussed here.

## Conclusions

**Figure 2:**
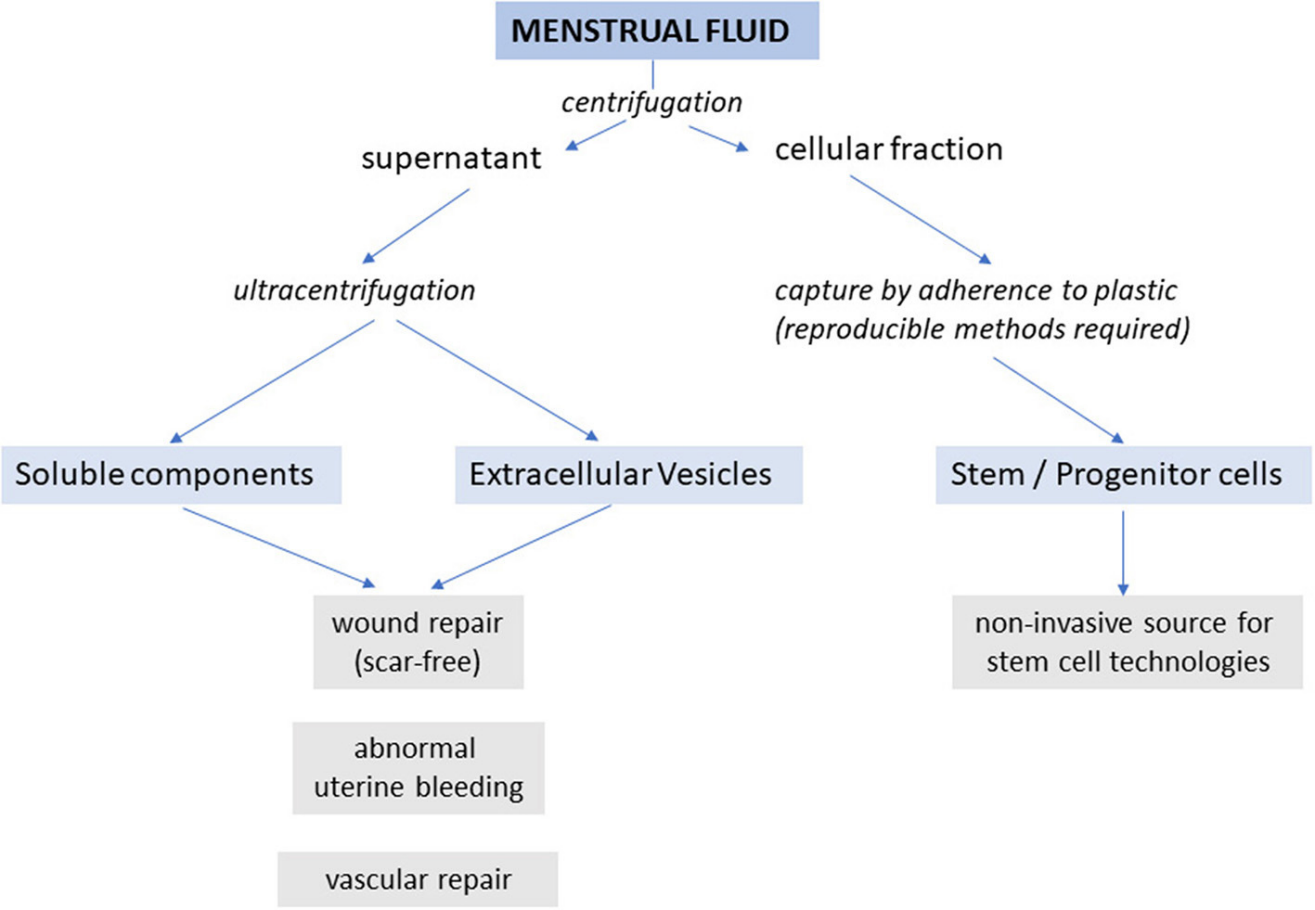
Schematic showing potential applications of components of menstrual fluid.

Rapid scar-free repair of the endometrium following menstruation is essential given that it occurs some 400 times during most women's reproductive lives and provides the basis for the subsequent regeneration and differentiation of the endometrium and its attainment of receptivity for embryo implantation in the new cycle. It is now indisputable that the microenvironment provided by menstrual fluid drives effective endometrial repair. Recent advances in analyses of menstrual fluid components has provided some insight but just which are the most important factors, soluble or those delivered to the damaged surface in extracellular vesicles, remains to be determined. Importantly menstrual fluid has the potential to provide factors for scar-free rapid wound repair, and to treat abnormal uterine bleeding (Figure 2).

## Author Contributions

The author confirms being the sole contributor of this work and has approved it for publication.

## Funding

LS and her laboratory have been funded extensively by Fellowships, Program and Project grants from the Australian National Health and Medical Research Council. Research at the Hudson Institute of Medical Research is funded in part by a State Government of Victoria Infrastructure support program.

## Conflict of Interest

The author declares that the research was conducted in the absence of any commercial or financial relationships that could be construed as a potential conflict of interest.

## Publisher's Note

All claims expressed in this article are solely those of the authors and do not necessarily represent those of their affiliated organizations, or those of the publisher, the editors and the reviewers. Any product that may be evaluated in this article, or claim that may be made by its manufacturer, is not guaranteed or endorsed by the publisher.
